# Tumor metabolic volume by ^18^F-FDG-PET as a prognostic predictor of first-line pembrolizumab for NSCLC patients with PD-L1 ≥ 50%

**DOI:** 10.1038/s41598-020-71735-y

**Published:** 2020-09-14

**Authors:** Ou Yamaguchi, Kyoichi Kaira, Kosuke Hashimoto, Atsuto Mouri, Ayako Shiono, Yu Miura, Yoshitake Murayama, Kunihiko Kobayashi, Hiroshi Kagamu, Ichiei Kuji

**Affiliations:** 1 Department of Respiratory Medicine, Comprehensive Cancer Center, International Medical Center, International Medical Center, Saitama Medical University, 1397-1 Yamane, Hidaka, Saitama 350-1298 Japan; 2 Department of Nuclear Medicine, Comprehensive Cancer Center, International Medical Center, Saitama Medical University, 1397-1 Yamane, Hidaka, Saitama 350-1298 Japan

**Keywords:** Cancer, Immunology, Oncology

## Abstract

There is a lack of markers for predicting favorable outcomes after pembrolizumab therapy in patients with non-small cell lung cancer (NSCLC) with programmed death ligand-1 (PD-L1) expression ≥ 50%. This retrospective study examined the prognostic significance of 2-deoxy-2-[18F] fluoro-d-glucose (^18^F-FDG) uptake as a predictive marker of first-line pembrolizumab. Forty-eight patients with previously untreated NSCLC and PD-L1 expression levels ≥ 50% who underwent ^18^F-FDG-positron emission tomography (PET) just before administration of pembrolizumab monotherapy were eligible and underwent assessment of metabolic tumor volume (MTV), total lesion glycolysis (TLG), and maximum of standardized uptake value (SUV_max_) on ^18^F-FDG uptake. The objective response rate, median progression-free survival, and median overall survival were 51.1%, 7.1 months, and 18.6 months, respectively. In univariate survival analyses, high MTV was barely a significant prognostic predictor and was confirmed as an independent factor linked to worse outcomes in multivariate analysis, predominantly in patients with a histological diagnosis of adenocarcinoma. A high MTV was significantly associated with distant metastases (especially bone metastasis), C-reactive protein (CRP) level, and PD-L1 expression ≥ 75%. Metabolic tumor activity assessed as MTV from ^18^F-FDG uptake predicted the prognosis after first-line pembrolizumab treatment in patients with NSCLC and PD-L1 expression ≥ 50%, especially for adenocarcinoma.

## Introduction

Immune checkpoint inhibitors (ICIs) are widely administered to patients with various kinds of neoplasms. In particular, anti-programmed death-1 (PD-1) or anti-programmed death ligand-1 (PD-L1) antibodies such as nivolumab, pembrolizumab, and atezolizumab show significant efficacy in patients with advanced non-small cell lung cancer (NSCLC). The varying efficacies of these antibodies according to PD-L1 expression within tumor cells have been reported^1–3^. Recent reports identified the combination therapy of platinum-based regimen plus pembrolizumab or atezolizumab as a standard first-line therapy in patients with advanced NSCLC, regardless of PD-L1 expression level^4,5^. As a first-line treatment in patients with PD-L1 expression ≥ 50%, however, it remains unclear whether the efficacy of the combination with platinum plus pembrolizumab is significantly superior to that of pembrolizumab monotherapy. While PD-L1 expression is clinically thought to be a unique biomarker for prediction of pembrolizumab efficacy, little is known about the established predictive markers in NSCLC patients with PD-L1 expression ≥ 50%^2^.

2-Deoxy-2-[^18^F] fluoro-d-glucose (^18^F-FDG) positron emission tomography (PET) is a convenient modality for the identification of cancer and tumor spread. The accumulation of ^18^F-FDG on PET suggests its use as a potential prognostic factor after treatments such as surgery or chemotherapy in patients with NSCLC^6–8^. A recent meta-analysis proposed that metabolic parameters such as total lesion glycolysis (TLG) and metabolic tumor volume (MTV) are better predictors of outcome after treatment compared to the maximum standardized uptake values (SUV_max_) in lung cancer^8^. We recently reported the clinical significance of therapeutic monitoring of anti-PD-1 antibody by ^18^F-FDG PET^9^ and the close relationship between ^18^F-FDG uptake and PD-L1 expression within tumor cells by immunohistochemistry^10–12^. Previous studies reported an association between PD-L1 expression and tumor glucose metabolism and hypoxia, while experimental investigation indicated that PD-L1 up-regulation is partially controlled by the expression of hypoxia-inducible factor 1α (HIF-1α)^13^. If the accumulation of ^18^F-FDG within tumor cells can be used to predict outcomes after initiation of anti-PD-1 antibody monotherapy, the usefulness and convenience of ^18^F-FDG PET make it a potential predictive factor for ICIs. In daily practice, PD-L1 expression is assessed by immunohistochemistry before initiation of anti-PD-1/PD-L1 therapy as a predictive marker; however, fewer than half of patients with PD-L1 expression ≥ 50% showed an objective response and approximately 20% experienced progressive disease^2^. More specific predictive markers are needed based on the results of clinic-based studies. Thus, it may be necessary to explore the established predictive markers of pembrolizumab in NSCLC patients with PD-L1 expression ≥ 50%.

Therefore, we retrospectively examined the prognostic significance of FDG-PET to predict response to first-line pembrolizumab monotherapy in patients with untreated advanced NSCLC with PD-L1 expression ≥ 50%.

## Results

### Patient demographics and PET study

This study enrolled a total of 48 patients (n_males_ = 39, n_females_ = 9; median age = 69 years; age range 47–86 years). Their demographics according to FDG uptake are listed in Table 1. A total of 43 patients (89%) had a smoking history. The performance status (PS) was 0, 1, 2, and 3 in 13 (27%), 17 (35%), 12 (25%), and 6 (13%) patients, respectively. Regarding clinical TNM staging, the patients with T1/T2/T3/T4 and N0/N1/N2/N3 were recognized in 7/13/8/20 and 5/4/15/24, respectively.

**Table 1:** Patient’s demographics according to ^18^F-FDG uptake.

Variables	Total patients	MTV			TLG			SUVmax		
	N = 48	High N = 12	Low N = 36	*p* value	High N = 21	High N = 27	*p* value	High N = 20	Low N = 28	*p* value
Age ≤ 69/> 69 years	25/23	7/5	18/18	0.74	10/11	15/12	0.77	9/11	16/12	0.55
Gender Male/female	39/9	10/2	29/7	> 0.99	17/4	22/5	> 0.99	17/3	22/6	0.71
Smoking Yes/no	43/5	11/1	32/4	> 0.99	19/2	24/3	> 0.99	19/1	24/4	0.38
PS 0–1/2–3	30/18	6/6	24/12	0.32	8/13	22/5	** < 0.01**	11/9	19/9	0.38
Histology AC/non-AC	23/25	3/9	20/16	0.09	5/16	18/9	** < 0.01**	6/14	17/11	**0.04**
Distant metastases Yes/no	37/11	12/0	25/11	**0.04**	21/0	16/11	** < 0.01**	17/3	20/8	0.31
ILD Yes/no	16/32	5/7	11/25	0.49	8/13	8/19	0.55	6/14	10/18	0.76
Previous radiation Yes/no	15/33	5/7	10/26	0.47	8/13	7/20	0.53	7/13	8/20	0.75
BMI High/low	22/26	4/8	18/18	0.50	8/13	14/13	0.39	8/12	14/14	0.56
NLR High/low	24/24	8/4	16/20	0.31	13/8	11/16	0.24	12/8	12/16	0.38
CRP ≤ 1.0/> 1.0	21/27	1/11	20/16	** < 0.01**	3/18	9/18	0.18	7/13	14/14	0.38
Response* Responder/non-responder	23/22	3/7	20/15	0.16	8/11	15/11	0.37	8/11	15/11	0.37
Brain metastases Yes/no	19/29	4/8	15/21	0.73	6/15	13/14	0.23	6/14	13/15	0.37
Bone metastasis Yes/no	17/31	8/4	9/27	**0.01**	13/8	4/23	** < 0.01**	12/8	5/23	** < 0.01**
Liver metastasis Yes/no	6/42	3/9	3/33	0.15	6/15	0/27	** < 0.01**	3/17	3/25	0.68
PD-L1 ≤75/> 75 (%)	25/23	3/9	22/14	**0.04**	16/5	9/18	** < 0.01**	14/6	11/17	**0.04**

*PS* performance status, *AC* adenocarcinoma, *ILD* interstitial lung disease, *BMI* body mass index, *NLR* neutrophil to lymphocyte ratio, *CRP* C-reactive protein, *TLG* total lesion glycolysis, *MTV* metabolic tumor volume, *SUVmax* the maximum of standardized uptake value, *PD-L1* programmed death ligand-1, Response* response rate was evaluated by RECIST(response evaluation criteria in solid tumors), and responder and non-responder were defined as CR(complete response) or PR(partial response) and SD(stable disease) or PD(progressive disease), respectively.

Bold means statistical significance.

The histology types included adenocarcinoma (Ad) in 23 (48%) patients, squamous cell carcinoma (SQC) in 11 (23%), and other in 14 (29%). Regarding PD-L1 expression, 25 (52%) and 23 (48%) patients had levels of 50–75% and ≥ 75%, respectively.

The median values for MTV, TLG, and SUV_max_ before pembrolizumab treatment were 112 cm^3^, 513 g cm^3^/mL, and 11, respectively. The optimal ^18^F-FDG uptake cut-offs for MTV, TLG, and SUV_max_ to differentiate responders from non-responders, as determined by receiver operating characteristic (ROC) curve analysis, were 268 cm^3^ (range 9–1,401, sensitivity: 91%, specificity: 32%), 697 g cm^3^/mL (range 33–7,475, sensitivity: 69%, specificity: 52%), and 11 (range 5–113, sensitivity: 65%, specificity: 48%), respectively. The area under the curve (AUC) for ROC analysis was 0.548 in MTV, 0.543 in TLG and 0.519 in SUV_max_. The median period between ^18^F-FDG PET and initiation of pembrolizumab was 27 days, ranging from 4 to 77 days. The median administration of pembrolizumab displayed 5 cycles (range 1–29 cycles), and the median follow-up period was 346 days, ranging from 30 to 884 days.

Table 1 shows the patients’ demographics according to ^18^F-FDG uptake. Assessment of ^18^F-FDG uptake showed that MTV was significantly associated with distant metastases, CRP level, bone metastases, and PD-L1 expression, while TLG was associated with PS, histology, distant metastases, and PD-L1 expression and SUV_max_ was associated with histology, bone metastases, and PD-L1 expression. Pearson rank tests showed that SUV_max_ on PET was significantly correlated with MTV (γ = 0.84, *p* < 0.001) and TLG (γ = 0.81, *p* < 0.001).

### Response and survival analysis

The overall median PFS and median OS were 7.1 and 18.6 months, respectively. Thirty-three patients experienced recurrences and 23 patients died from progressive disease. Among the 48 patients, 23, 12, 10 patients exhibited PR, SD, and PD, respectively. No patients showed a CR and three patients were not evaluated (NE). The objective response rate was 51.1% (95% confidence interval 36.5–65.7%). Analysis of ^18^F-FDG uptake showed objective response rates in patients with high and low MTV, high and low TLG, and high and low SUV_max_ of 30% (3/10) and 57% (20/35) (p = 0.16), 42% (8/19) and 57% (15/26) (*p* = 0.37), and 42% (8/19) and 57% (15/26) (*p* = 0.37), respectively.

Aside from the three NE patients, 45 patients were analyzed according to responder or non-responder status (Table 2). High body mass index (BMI) was statistically associated with responders. The analysis on the relationship between response and PFS and OS was performed, and the PFS and OS were significantly better in patients with CR or PR than in those with SD or PD.

**Table 2:** Patient’s demographics according to responder or non-responder.

Variables	Total patients	Responder	Non-responder	*p* value
	N = 45	N = 23	N = 22	
Age ** ≤ **69/> 69 years	24/21	13/10	11/11	0.76
Gender Male/female	36/9	20/3	16/6	0.28
Smoking Yes/no	41/4	20/3	21/1	0.61
PS 0–1/2–3	29/16	15/8	14/8	> 0.99
Histology AC/non-AC	22/23	10/13	12/10	0.76
Distant meta Yes/no	34/11	16/7	18/4	0.49
ILD Yes/no	15/30	6/17	9/13	0.35
Previous radiation Yes/no	14/31	5/18	9/13	0.21
BMI High/low	20/25	14/9	6/16	**0.03**
NLR High/low	22/23	12/11	10/12	0.76
CRP ** ≤ **1.0/> 1.0	20/25	12/11	8/14	0.37
SUVmax High/low	19/26	8/15	11/11	0.37
MTV High/low	10/35	3/20	7/15	0.16
TLG High/low	19/26	8/15	11/11	0.37
PD-L1 ** ≤ **75/> 75 (%)	24/21	12/11	12/10	> 0.99

*PS* performance status, *AC* adenocarcinoma, *ILD* interstitial lung disease, *BMI* body mass index, *NLR* neutrophil to lymphocyte ratio, *CRP* C-reactive protein, *TLG* total lesion glycolysis, *MTV* metabolic tumor volume, *SUVmax* the maximum of standardized uptake value, *PD-L1* programmed death ligand-1.

Bold means statistical significance.

Univariate survival analyses were performed using variables including MTV, TLG, SUV_max_, age, sex, smoking, performance status, histology, distant metastases (brain, bone, and liver), history of previous irradiationn, BMI, neutrophil-to-lymphocyte ratio, PD-L1 expression level (< 75% vs. ≥ 75%), and C-reactive protein (CRP) level. Only high MTV was a marginally significant factor for predicting worse OS, but not PFS (Table 3) and was identified as an independent factor linked to worse OS in multivariate analysis (Table 4).

**Table 3:** Univariate survival analysis.

Variables	PFS (MST: days)	p value	OS (MST: days)	p value
Age ** ≤ **69/> 69 years	196/216	0.50	NR/437	0.21
Gender Male/female	240/203	0.46	637/490	0.38
Smoking Yes/no	240/196	0.93	637/490	0.63
PS 0–1/2–3	324/168	0.18	568/324	0.54
Histology AC/non-AC	388/167	0.06	637/317	0.05
Distant meta Yes/no	203/361	0.75	437/568	0.51
ILD Yes/no	388/196	0.39	490/568	0.87
Extracranial radiation Yes/no	196/240	0.93	568/637	0.36
BMI High/low	196/240	0.45	779/490	0.31
NLR High/low	196/216	0.66	NR/568	0.77
CRP ** ≤ **1.0/> 1.0	361/167	0.11	568/437	0.21
SUVmax High/low	164/244	0.60	568/490	0.91
MTV High/low	104/244	0.32	124/637	**0.04**
TLG High/low	167/324	0.21	203/568	0.13
PD-L1 ** ≤ **75/> 75 (%)	324/203	0.52	568/490	0.73
Brain metastases Yes/no	456/196	0.07	637/437	0.64
Bone metastasis Yes/no	203/296	0.86	437/568	0.87
Liver metastasis Yes/no	65/240	0.58	114/568	0.10

*PFS* progression free survival, *OS* overall survival, *MST* median survival time, *PS* performance status, *AC* adenocarcinoma, *ILD* interstitial lung disease, *BMI* body mass index, *NLR* neutrophil to lymphocyte ratio, *CRP* C-reactive protein, *TLG* total lesion glycolysis, *MTV* metabolic tumor volume, *SUVmax* the maximum of standardized uptake value, *PD-L1* programmed death ligand-1.

Bold means statistical significance.

**Table 4:** Multivariate Survival Analysis in all patients (n = 48).

Variables	PFS		OS	
	HR 95% CI	*p* value	HR 95% CI	*p* value
Age ** ≤ **69/> 69 years	0.66 0.31–1.35	0.25	0.55 0.22–1.29	0.18
Gender Male/female	1.51 0.58–3.47	0.35	1.42 0.79–2.33	0.18
PS 0–1/2–3	1.64 0.77–3.40	0.18	1.14 0.74–1.72	0.52
MTV High/low	1.49 0.77–3.24	0.32	1.57 0.98–2.41	**0.04**

*PFS* progression free survival, *OS* overall survival, *HR* hazard ratio, *95% CI* 95% confidence interval, *PS* performance status, *MTV* metabolic tumor volume.

Bold means statistical significance.

**Figure 1:**
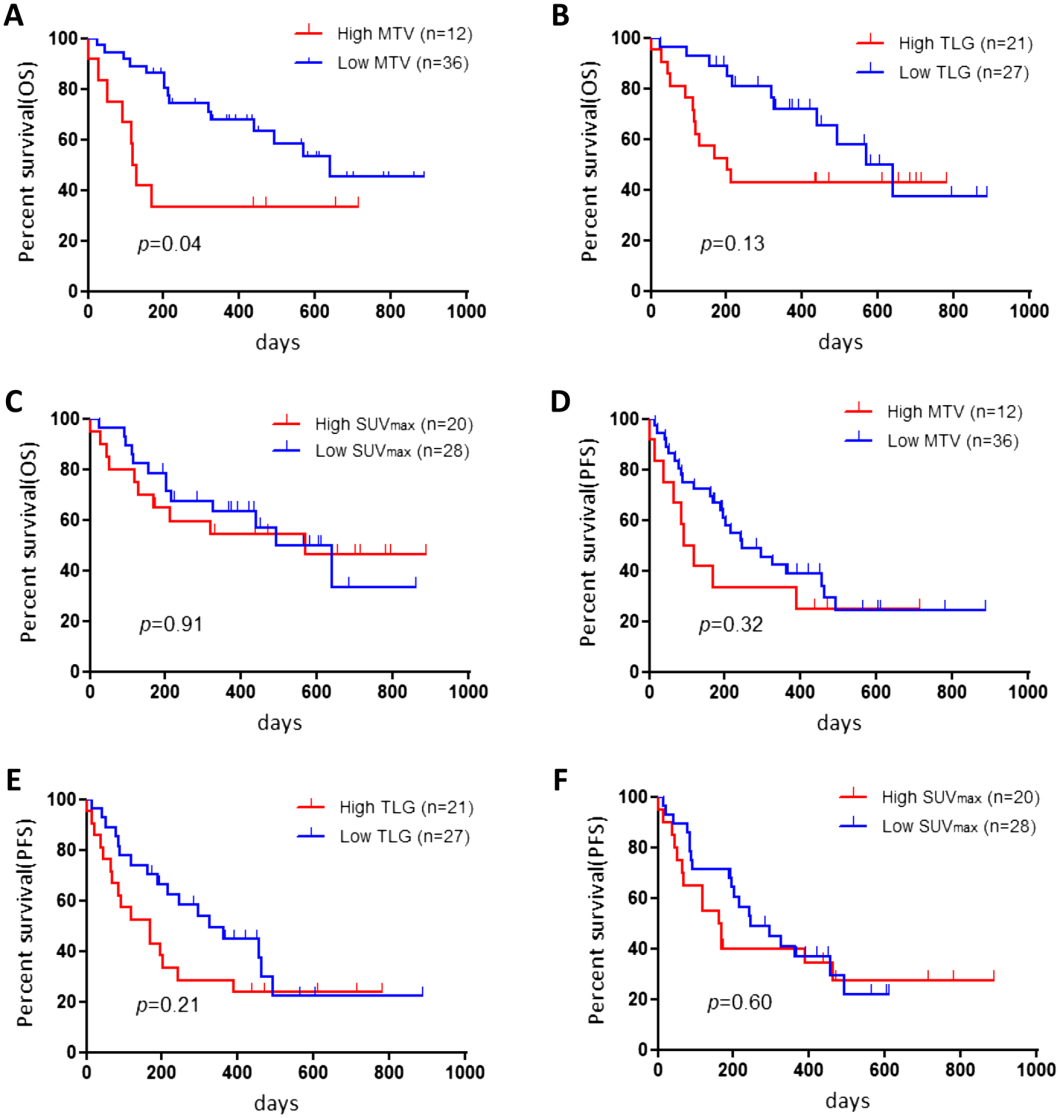
Kaplan–Meier curves according to various metabolic tumor volumes (MTVs) (**A**,**D**), total lesion glycolysis (TLG) (**B**,**E**), and maximum of standardized uptake value (SUVmax) (**C**,**F**) for overall survival (OS) and progression-free survival (PFS) in all patients. Patients with high MTV exhibited a significantly worse PFS (**A**), but not OS (**D**), than those with low MTV. No statistically significant differences in the PFS and OS were observed between patients with high and low TLG (**B**,**F**) and with high and low SUVmax (**C**,**F**).

**Figure 2:**
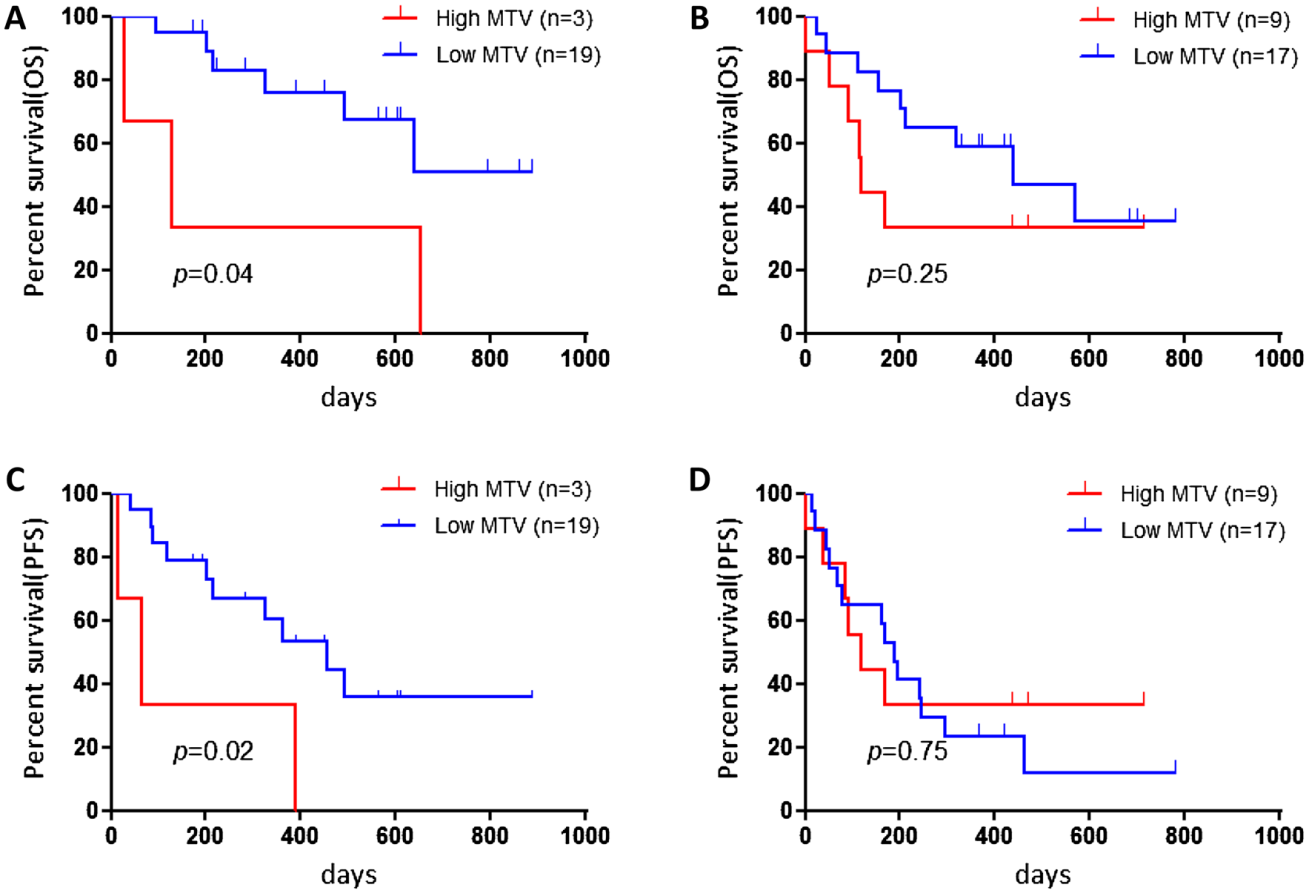
Kaplan–Meier curves according to various metabolic tumor volumes (MTVs) for overall survival (OS) and progression-free survival (PFS) in patients with adenocarcinoma (**A**,**B**) and non-adenocarcinoma (**C**,**D**). Adenocarcinoma patients with high MTV showed significantly worse PFS and OS than those in patients with low MTV (**A**,**B**). No statistically significant differences in PFS and OS were observed between non-adenocarcinoma patients with high and low MTV (**C**,**D**).

Next, we analyzed the survival information according to the histological types of adenocarcinoma (Ad) and non-Ad. In patients with Ad (n = 21), we observed a statistically significant difference in OS and PFS between those with low and high MTV but not with TLG and SUVmax. However, we observed no statistically significant difference in OS and PFS in patients with non-Ad between SUVmax, MTV, and TLG and ^18^F-FDG uptake. Kaplan–Meier survival curves are shown in Figs. 1 and 2.

## Discussion

To our knowledge, this is the first study to show that metabolic tumor activity, as assessed by MTV, could predict outcome in patients with advanced NSCLC with PD-L1 expressions ≥ 50%. In particular, metabolic activity on PET was closely associated with outcome after pembrolizumab treatment in patients histologically diagnosed with Ad but not in those without Ad. High MTV was significantly associated with distant metastases (especially, bone metastasis), CRP, and PD-L1 expression ≥ 75%.

PD-L1 expression level is considered to be the most useful marker for predicting the efficacy following ICI administration in patients with advanced NSCLC. While researchers continue to explore promising biomarkers as predictors of response to ICIs, nothing has yet been reported aside from PD-L1. Recently, Amrane et al*.* reported the efficacy and safety profile of first-line pembrolizumab for NSCLC patients with PD-L1 expression ≥ 50% in a multicenter real-life cohort^15^. Their retrospective study observed a median PFS of 10.1 months and an objective response rate of 57.3% in 108 patients, demonstrating similar PFS to the pivotal clinical study. In real-world data, Morita et al. reported that steroid use before treatment, malignant pleural effusion, and baseline CRP levels > 1.0 mg/dL decreased the response to first-line pembrolizumab in NSCLC patients with PD-L1 expression ≥ 50%^16^. The relationship between steroid use and anti-PD-1 antibody efficacy was reported previously, in which ≥ 10 mg prednisone at baseline was related to a worse prognosis in patients with NSCLC administered PD-1 blockade^17^. Likewise, elevated CRP level (> 1.0 mg/dL) and malignant pleural effusion were previously identified as negative predictors of PD-1 blockade^17,18^. Therefore, the three factors described by Morita et al. did not greatly add to our knowledge of predictive markers for first-line pembrolizumab^16^.

We recently reported that metabolic tumor activity on ^18^F-FDG PET is effective for differentiating responders from non-responders to anti-PD-1 antibody at the earliest phases (2 weeks or 1 month) after initiation^9,19^. Seith et al*.* demonstrated that ^18^F-FDG PET could potentially identify complete responders to anti-PD-1 antibody therapy as early as 2 weeks after its initial administration in patients with malignant melanoma^19^. We also described that metabolic tumor response on ^18^F-FDG was useful for predicting efficacy and survival at 1 month after anti-PD-1 antibody therapy in patients with previously treated NSCLC^9^. Although the prognostic significance of therapeutic monitoring as early phase after first-line pembrolizumab remains unclear, the potential of ^18^F-FDG PET as a therapeutic response to pembrolizumab requires further investigation in a prospective study. In the present study, ^18^F-FDG uptake before pembrolizumab initiation did not differ between patients with and without tumor shrinkage (PR versus SD or PD); however, this result is controversial^20^. Takada et al. examined the relationship between the response to anti-PD-1 antibody and ^18^F-FDG uptake before its initiation in 89 patients with recurrent or advanced NSCLC and found a significantly higher response rate of patients with increased SUVmax than that in those with decreased SUVmax^20^. However, they did not identify SUVmax as a significant factor for predicting the outcome of anti-PD-1 antibody, in line with our findings^20^. Previous clinicopathological studies demonstrated that PD-L1 expression level within tumor cells was closely correlated with ^18^F-FDG uptake^10–12^; therefore, the accumulation of ^18^F-FDG may be expected to predict the efficacy of anti-PD-1 antibody, similar to the role of PD-L1 expression as a predictive marker. In patients with Burkitt’s lymphoma as an aggressive lymphoma, it has reported that metabolic tumor volumes determined by MTV and TLG significantly correlated with response to treatment and long-term survival compared to SUV_max_^21^. Also, Albano et al. reported that MTV and TLG were closely associated with therapeutic response and PFS in patients with mantle cell lymphoma^22^.

Considering these controversial results, the relationship between anti-PD-1 antibody response and ^18^F-FDG uptake remains unclear. While little is known about the correlation between the therapeutic response to PD-1 blockade and metabolic tumor activity such as MTV or TLG, our results support the potential of ^18^F-FDG uptake as a predictive factor of its therapeutic response.

It is unclear why metabolic tumor activity as assessed by MTV can predict the outcome of first-line pembrolizumab in patients with PD-L1 expression ≥ 50%. There is no rationale to explain the mechanism of metabolic tumor activity as a prognostic marker of PD-1 blockade; however, a meta-analysis also identified metabolic parameters such as MTV and TLG as better prognostic markers in lung cancer^8^. Since metabolic tumor activity is considered to concisely reflect the tumor volume, high HIF-1 expression caused by increased tumor size may create an immunosuppressive environment in which Foxp3-regulatory T-cells (Tregs) are reported to be positively associated with MTV^23–25^. We speculate that the increased MTV forms a negative tumor immune microenvironment that is resistant to PD-1 blockade regardless of increased PD-L1 expression. Further studies with larger sample sizes are warranted to further elucidate our results.

This study has several limitations. First, our sample size is very limited and lack of a control group, which may bias the results. Thus, our study is an exploratory investigation and need more additional sample size for the confirmation of the results of the present study. Second, our approach focused on the clinical and prognostic significance of metabolic tumor activity according to ^18^F-FDG uptake. As ^18^F-FDG uptake is closely correlated with glucose transporter 1 (Glut1), HIF-1α, and PD-L1 expression, whether immunohistochemical markers such as GLUT1 and HIF-1α can predict outcome after first-line pembrolizumab treatment should be assessed. Further study by immunohistochemistry should be performed using tumor specimens.

In our study, moreover, the median period between ^18^F-FDG PET and initiation of pembrolizumab was 27 days (range 4–77 days). Three patients were treated with pembrolizumab at approximately 2 months after ^18^F-FDG-PET work. The lead time bias may be concern as a confound factor to affect the prognostic value of MTV.

Finally, we did not present data on first-line pembrolizumab treatment in patients with PD-L1 expression < 50%. Thus, the differences in the prognostic role of metabolic tumor activity according to PD-L1 expression remains unknown. We observed, no statistically significant difference in prognosis between patients with PD-L1 expression levels of 50–75% and ≥ 75%; however, the prognostic significance of tumor metabolic activity should be examined in patients with expression levels < 50%.

In conclusion, metabolic tumor activity as MTV on ^18^F-FDG uptake could predict the prognosis after first-line pembrolizumab treatment in patients with NSCLC with PD-L1 expression ≥ 50%, especially for patients with Ad. We identified glucose metabolism within tumor cells as a promising predictor for PD-1 antibody therapy. Further study is warranted to elucidate the prognostic significance of metabolic tumor activity measured as MTV in patients with advanced NSCLC who are candidates for combination therapy including ICIs.

## Patients and methods

### Patients

We examined the medical records of 63 patients with previously untreated NSCLC who underwent FDG-PET before initiation of pembrolizumab monotherapy in our institution between December 2017 and June 2019. The inclusion criteria for pembrolizumab treatment was defined as chemo-naïve NSCLC patients with PD-L1 expression levels ≥ 50%. Of these 63 patients, 15 were excluded for PD-L1 expression levels < 50%. Thus, 48 patients were eligible for analysis. All procedures performed in studies involving human participants were in accordance with the ethical standards of the institutional and/or national research committee and with the 1964 Declaration of Helsinki and its later amendments or comparable ethical standards. This study was approved by the Institutional Ethics Committee of the Saitama Medical University International Medical Center. Informed consent: The requirement for written informed consent (The ethics committee of Saitama Medical University) was waived because of the retrospective nature of the study.

### Treatment and evaluation

Pembrolizumab was intravenously administered at 200 mg/kg every 3 weeks. Physical examination, complete blood cell count, biochemical testing, and adverse events assessment were done triweekly according to the judgment of each chief physician. Acute toxicity was graded using the Common Terminology Criteria for Adverse Events version 4.0. Tumor response was evaluated using the Response Evaluation Criteria in Solid Tumors version 1.1^14^.

### PET imaging and data analysis

Patients fasted for at least 6 h before the performance on a PET/computed tomography (CT) scanner (Biograph 6 or 16, Siemens Healthineers K.K., Japan) with a 585 mm field of view. The acquisition of three-dimensional data was initiated 60 min after injecting 3.7 MBq/kg FDG. Eight bed positions (2-min acquisition per bed position) were obtained according to the range of PET imaging. Attenuation-corrected transverse images getting from ^18^F-FDG were reconstructed with the ordered-subsets expectation–maximization algorithm based on the point spread function into 168 × 168 matrices with a 2.00-mm slice thickness.

By semiquantitative analysis, functional images of SUV were provided by attenuation-corrected transaxial images, injected dosage of ^18^F-FDG, patient’s body weight, and the cross-calibration factor between PET and the dose calibrator. The SUV was defined as follows:

$${\text{SUV}} = {\text{Radioactive concentration in the volume of interest }}({\text{VOI}}) \, \left[ {\text{MBq/g}} \right]{\text{/Injected dose (MBq)/Patient}} {\text{'s body weight (g)}}.$$

A nuclear physician conducted VOI analysis using CT scans, eliminating the physiological uptake in the heart and urinary and gastrointestinal tracts. We used GI-PET software (Nihon Medi-physics Co. Ltd., Japan) on a Windows workstation to semi-automatically calculate the MTV and TLG (= SUV_mean_ × MTV), of each lesion using SUV thresholds in the liver VOI (= SUV_mean_ + [1.5 × SUV_Standard_Deviation_]). These SUV thresholds were the optimum values to generate VOIs in which the whole tumor mass was completely enclosed in all cases, with the CT image as the reference. SUV_max_ within the generated VOI were also calculated automatically. Measurable lesions were defined as FDG accumulation with more than 1.5 times the liver average uptake and the metabolic volume of 1 cm^3^ or more. Then, the uptake in normal structures such as the brain, tonsils, heart, intestinal tract, stomach, gastrointestinal tract, renal urinary tract system, was manually excluded by the visual inspection. VOIs over all measurable lesions on pretreatment PET/CT were automatically registered. In cases with multiple lesions in the same organ, a maximum of 100 lesions were measured. This PET analysis was performed according to that of previous study^26^.

### Statistical analysis

Statistical significance was indicated by p < 0.05. Fisher’s exact tests were used to examine the association between two categorical variables. Correlations between SUVmax, MTV, and TLG on ^18^F-FDG uptake were analyzed using Pearson rank tests. The Kaplan–Meier method was used to estimate survival as a function of time and survival differences were analyzed by log-rank tests. Progression-free survival (PFS) was defined as the time from anti-PD-1 antibody initiation to tumor recurrence or death from any cause, while overall survival (OS) was defined as the time from anti-PD-1 antibody initiation to death from any cause. ROC analyses were performed according to the previous study^9^. Responder was defined as a complete response (CR) or partial response (PR) with a PFS greater than 6 months. Survival time was a continuous variable for ROC analysis. Statistical analyses were performed using GraphPad Prism 7 (Graph Pad Software, San Diego, CA, USA) and JMP 14.0 (SAS Institute Inc., Cary, NC, USA). This analysis was done according to that of previous study^26^.

### Ethical approval

All procedures performed in studies involving human participants were in accordance with the ethical standards of the institutional and/or national research committee and with the 1964 Declaration of Helsinki and its later amendments or comparable ethical standards.

### Informed consent

The requirement for written informed consent (The ethics committee of Saitama Medical University) was waived because of the retrospective nature of the study.
